# 2,4-Di­bromo-1,3-dihy­droxy-9*H*-xanthen-9-one

**DOI:** 10.1107/S1600536813019296

**Published:** 2013-07-31

**Authors:** Shi-Wen Huang, Zheng-Min Yang, Fu-Ping Huang, Jiang-Ke Qin

**Affiliations:** a Key Laboratory for the Chemistry & Molecular Engineering of Medicinal Resources, Ministry of Education of China, School of Chemistry & Chemical Engineering, Guangxi Normal University, Guilin, 541004, People’s Republic of China; b Department of Pharmacy, Youjiang Medical University for Nationalities, Baise 533000, People’s Republic of China

## Abstract

The title compound, C_13_H_6_Br_2_O_4_, derived from xanthone, a fundamental structural framework of active ingredients in many medicinal plants, and was synthesized by bromination of 1,3-di­hydroxyxanthen-9-one with *N*-bromo­succinimide. The mol­ecular conformation is essentially planar, the dihedral angle between the benzene rings being 1.1 (4)°. This conformation is favorable for the formation of an intra­molecular O—H⋯O hydrogen bond between a hy­droxy group and the xanthone carbonyl group. In the crystal, mol­ecules are associated into chains along the *b*-axis direction *via* C=O⋯H—O hydrogen bonds involving the other hy­droxy group.

## Related literature

For the pharmacological activity of xanthone derivatives, see: Cheng *et al.* (2011 ▶); Dao *et al.* (2012 ▶); Sousa *et al.* (2009 ▶); Szkaradek *et al.* (2013 ▶). For the synthesis of the xanthone used as a starting material, see: Liu *et al.* (2006 ▶). For related xanthone structures, see: Corrêa *et al.* (2010 ▶).
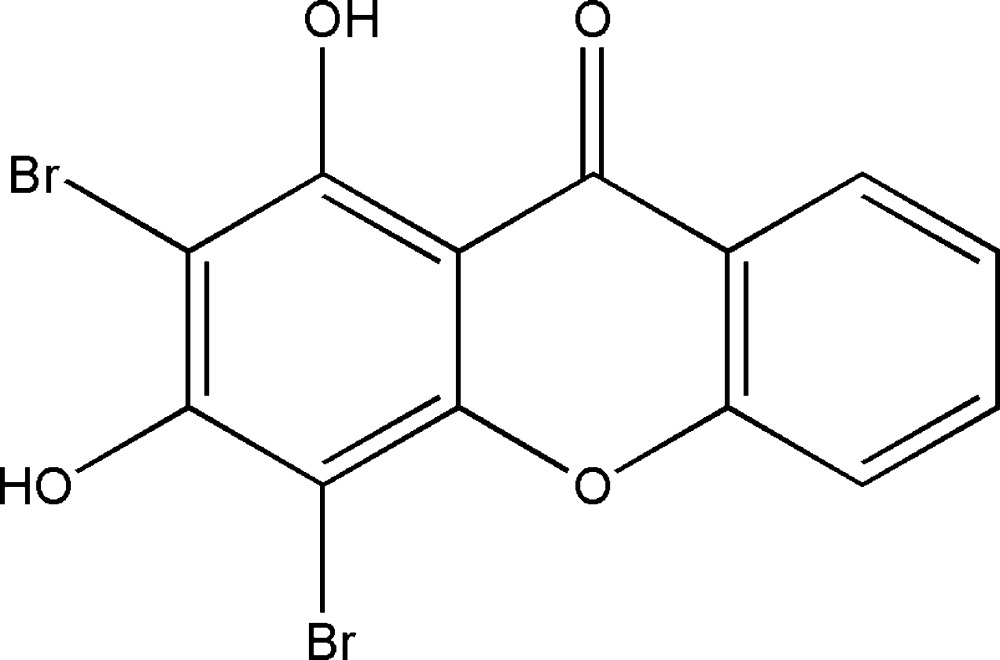


## Experimental

### 

#### Crystal data

C_13_H_6_Br_2_O_4_
*M* *_r_* = 386.00
Orthorhombic, 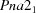

*a* = 18.4489 (15) Å
*b* = 16.9049 (13) Å
*c* = 3.8564 (3) Å
*V* = 1202.72 (16) Å^3^
*Z* = 4
Mo *K*α radiation
μ = 6.75 mm^−1^
*T* = 298 K
0.28 × 0.09 × 0.06 mm

#### Data collection

Bruker SMART CCD diffractometer
Absorption correction: multi-scan (*SADABS*; Bruker, 1998 ▶) *T*_min_ = 0.254, *T*_max_ = 0.688
6188 measured reflections
2120 independent reflections
1830 reflections with *I* > 2σ(*I*)
*R*_int_ = 0.076

#### Refinement

*R*[*F* ^2^ > 2σ(*F* ^2^)] = 0.034
*wR*(*F* ^2^) = 0.070
*S* = 1.04
2120 reflections
172 parameters
1 restraint
H-atom parameters constrained
Δρ_max_ = 0.43 e Å^−3^
Δρ_min_ = −0.33 e Å^−3^
Absolute structure: Flack (1983 ▶), 881 Friedel pairs
Absolute structure parameter: −0.008 (16)

### 

Data collection: *SMART* (Bruker, 1998 ▶); cell refinement: *SAINT* (Bruker, 1998 ▶); data reduction: *SAINT* (Bruker, 1998 ▶); program(s) used to solve structure: *SHELXS97* (Sheldrick, 2008 ▶); program(s) used to refine structure: *SHELXL97* (Sheldrick, 2008 ▶); molecular graphics: *SHELXL97* (Sheldrick, 2008 ▶); software used to prepare material for publication: *SHELXL97* (Sheldrick, 2008 ▶).

## Supplementary Material

Crystal structure: contains datablock(s) I, New_Global_Publ_Block. DOI: 10.1107/S1600536813019296/bh2480sup1.cif

Structure factors: contains datablock(s) I. DOI: 10.1107/S1600536813019296/bh2480Isup2.hkl

Supplementary material file. DOI: 10.1107/S1600536813019296/bh2480Isup3.cdx

Supplementary material file. DOI: 10.1107/S1600536813019296/bh2480Isup4.cml

Additional supplementary materials:  crystallographic information; 3D view; checkCIF report

## Figures and Tables

**Table 1:** Hydrogen-bond geometry (Å, °)

*D*—H⋯*A*	*D*—H	H⋯*A*	*D*⋯*A*	*D*—H⋯*A*
O4—H4⋯O2	0.82	1.81	2.555 (7)	149
O3—H3⋯O2^i^	0.82	2.02	2.741 (7)	147

Symmetry code: (i) 
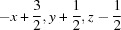
.

**Fig. 1.**
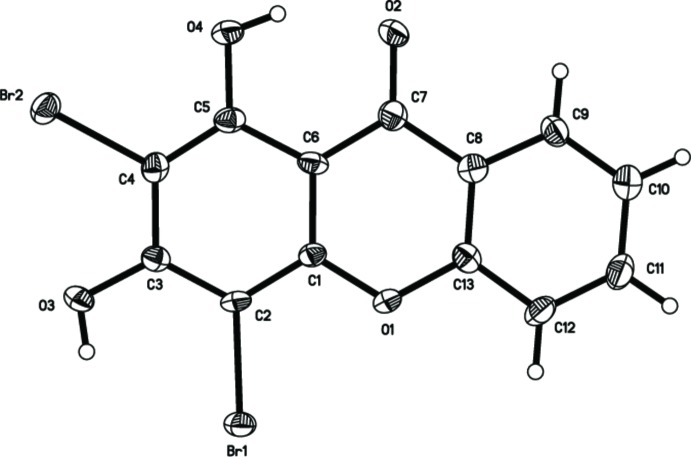
The structure of the title compound and the atom-numbering scheme.

**Fig. 2.**
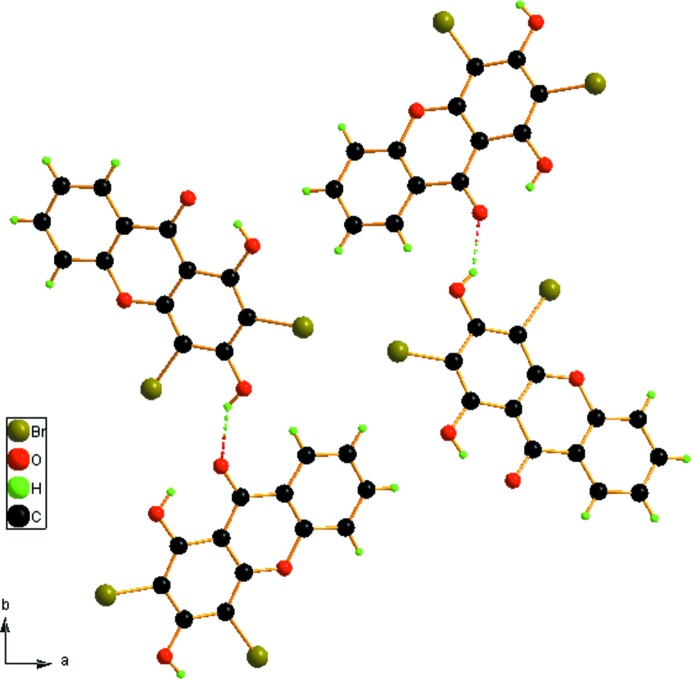
The packing diagram of the title compound.

## supplementary crystallographic information

### Comment

Xanthone, also named as dibenzo-γ-pyrone, is a fundamental structural framework of active ingredients in many medicinal plants, which derivatives have broad pharmacological activities, such as antioxidant (Cheng *et al.*, 2011), antitumor (Sousa *et al.*, 2009), anticonvulsant (Szkaradek *et al.*, 2013) and inhibition of neuraminidase activity (Dao *et al.*, 2012). The title compound in this study is a new xanthone derivative, which was synthesized by bromination of 1,3-dihydroxy-xanthen-9-one (Liu *et al.*, 2006) with NBS.

We report here the synthesis and crystal structure of 2,4-dibromo-1,3-dihydroxy-xanthen-9-one (C_13_H_6_Br_2_O_4_, Fig. 1). The molecule of the title compound has a planar conformation, with characteristic bond lengths C2—Br1 = 1.893 (5) Å and C4—Br2 = 1.902 (5) Å. The molecular conformation is mainly controlled by the O4—H4···O2 intramolecular hydrogen bond between the hydroxy OH group and the carbonyl O atom. Molecules are further connected into a one-dimensional supramolecular architecture *via* O3—H3···O2 intermolecular hydrogen bonds (Fig. 2). The title compound has the same planar molecular conformation as that reported for other 1-hydroxy-9*H*-xanthen-9-one derivatives (Corrêa *et al.*, 2010), while the carbonyl bond length C7═O2, 1.262 (7) Å, is slightly larger than the corresponding carbonyl bond lengths in these derivatives.

### Experimental

The title compound was synthesized using the following procedure: in a 50 ml flask, 1,3-dihydroxy-xanthen-9-one (1010 mg, 4.43 mmol; Liu *et al.*, 2006) was dissolved in CCl_4_ (15 ml), then NBS (500 mg, 2.83 mmol) was added. The mixture was stirred at room temperature for 24 h. The residue was washed with acetone and then filtered. The yellow solid was collected and dried. Recrystallization from methanol solution afforded 2,4-dibromo-1,3-dihydroxy-xanthen-9-one as yellow crystals. The compound identity was confirmed by NMR spectroscopy. ^1^H NMR (500 MHz, DMSO-*d*_6_): 13.63 (*s*, 1H), 8.09 (*dd*, *J* = 7.9, 1.6 Hz, 1H), 7.87 (*td*, *J* = 7.8, 1.5 Hz, 1H), 7.59 (*d*, *J* = 8.4 Hz, 1H), 7.48 (*t*, *J* = 7.5 Hz, 1H).

### Refinement

The H atoms on C and O atoms were positioned geometrically and refined using a riding model, with C—H = 0.93 Å and *U*_iso_(H) = 1.2*U*_eq_(C), and with O—H = 0.82 Å and *U*_iso_(H) = 1.5*U*_eq_(O).

### Crystal data

**Table:**

C_13_H_6_Br_2_O_4_	*F*(000) = 744
*M**_r_* = 386.00	*D*_x_ = 2.132 Mg m^−^^3^
Orthorhombic, *P**n**a*2_1_	Mo *K*α radiation, λ = 0.71073 Å
Hall symbol: P 2c -2n	Cell parameters from 2778 reflections
*a* = 18.4489 (15) Å	θ = 3.3–26.2°
*b* = 16.9049 (13) Å	µ = 6.75 mm^−^^1^
*c* = 3.8564 (3) Å	*T* = 298 K
*V* = 1202.72 (16) Å^3^	Block, yellow
*Z* = 4	0.28 × 0.09 × 0.06 mm

### Data collection

**Table:**

Bruker SMART CCD diffractometer	2120 independent reflections
Radiation source: fine-focus sealed tube	1830 reflections with *I* > 2σ(*I*)
Graphite monochromator	*R*_int_ = 0.076
φ and ω scans	θ_max_ = 25.0°, θ_min_ = 1.6°
Absorption correction: multi-scan (*SADABS*; Bruker, 1998)	*h* = −21→21
*T*_min_ = 0.254, *T*_max_ = 0.688	*k* = −20→15
6188 measured reflections	*l* = −4→4

### Refinement

**Table:**

Refinement on *F*^2^	Secondary atom site location: difference Fourier map
Least-squares matrix: full	Hydrogen site location: inferred from neighbouring sites
*R*[*F*^2^ > 2σ(*F*^2^)] = 0.034	H-atom parameters constrained
*wR*(*F*^2^) = 0.070	*w* = 1/[σ^2^(*F*_o_^2^) + (0.0232*P*)^2^] where *P* = (*F*_o_^2^ + 2*F*_c_^2^)/3
*S* = 1.04	(Δ/σ)_max_ = 0.002
2120 reflections	Δρ_max_ = 0.43 e Å^−^^3^
172 parameters	Δρ_min_ = −0.33 e Å^−^^3^
1 restraint	Absolute structure: Flack (1983), 881 Friedel pairs
0 constraints	Flack parameter: −0.008 (16)
Primary atom site location: structure-invariant direct methods	

### Fractional atomic coordinates and isotropic or equivalent isotropic displacement parameters (Å^2^)

**Table:**

	*x*	*y*	*z*	*U*_iso_*/*U*_eq_	
Br1	0.65912 (3)	1.09241 (3)	−0.07056 (16)	0.03711 (16)	
Br2	0.91885 (3)	0.97740 (3)	0.54899 (17)	0.03972 (17)	
O1	0.61335 (17)	0.9267 (2)	0.0466 (12)	0.0366 (9)	
O2	0.72160 (18)	0.7368 (2)	0.4658 (13)	0.0483 (11)	
O3	0.8177 (2)	1.0920 (2)	0.2248 (11)	0.0408 (11)	
H3	0.7904	1.1248	0.1377	0.061*	
O4	0.83232 (18)	0.8253 (2)	0.5513 (13)	0.0452 (11)	
H4	0.8074	0.7851	0.5521	0.068*	
C1	0.6824 (3)	0.9348 (3)	0.1582 (13)	0.0262 (13)	
C2	0.7137 (3)	1.0086 (3)	0.1241 (13)	0.0277 (13)	
C3	0.7844 (3)	1.0223 (3)	0.2401 (14)	0.0293 (13)	
C4	0.8233 (3)	0.9589 (3)	0.3828 (15)	0.0310 (14)	
C5	0.7941 (3)	0.8853 (3)	0.4141 (16)	0.0323 (13)	
C6	0.7210 (3)	0.8704 (3)	0.2998 (14)	0.0305 (14)	
C7	0.6879 (3)	0.7943 (4)	0.3340 (15)	0.0351 (15)	
C8	0.6131 (3)	0.7875 (3)	0.2015 (14)	0.0337 (14)	
C9	0.5753 (3)	0.7164 (4)	0.2048 (17)	0.0400 (15)	
H9	0.5977	0.6707	0.2858	0.048*	
C10	0.5053 (3)	0.7135 (4)	0.0887 (19)	0.0479 (16)	
H10	0.4797	0.6661	0.0978	0.057*	
C11	0.4724 (3)	0.7802 (4)	−0.0412 (18)	0.0458 (15)	
H11	0.4251	0.7770	−0.1236	0.055*	
C12	0.5080 (3)	0.8511 (3)	−0.0515 (17)	0.0410 (14)	
H12	0.4853	0.8965	−0.1333	0.049*	
C13	0.5793 (3)	0.8533 (3)	0.0642 (17)	0.0314 (12)	

### Atomic displacement parameters (Å^2^)

**Table:**

	*U*^11^	*U*^22^	*U*^33^	*U*^12^	*U*^13^	*U*^23^
Br1	0.0367 (3)	0.0309 (3)	0.0438 (3)	0.0061 (2)	−0.0024 (3)	0.0080 (3)
Br2	0.0277 (3)	0.0461 (4)	0.0454 (3)	−0.0007 (2)	−0.0040 (3)	0.0016 (3)
O1	0.0249 (19)	0.033 (2)	0.052 (2)	0.0044 (15)	−0.007 (2)	0.008 (2)
O2	0.037 (2)	0.027 (2)	0.081 (3)	0.0009 (17)	−0.006 (3)	0.015 (2)
O3	0.040 (2)	0.025 (2)	0.058 (3)	−0.0020 (19)	−0.005 (2)	0.012 (2)
O4	0.032 (2)	0.037 (2)	0.067 (3)	0.0062 (16)	−0.010 (3)	0.015 (2)
C1	0.025 (3)	0.026 (3)	0.027 (3)	0.006 (2)	0.000 (2)	−0.002 (2)
C2	0.028 (3)	0.028 (3)	0.026 (4)	0.012 (2)	−0.002 (2)	0.001 (2)
C3	0.028 (3)	0.028 (3)	0.032 (3)	0.003 (3)	0.005 (2)	0.003 (3)
C4	0.022 (3)	0.036 (3)	0.034 (3)	0.000 (2)	0.006 (3)	−0.002 (3)
C5	0.027 (3)	0.036 (3)	0.034 (3)	0.006 (2)	0.005 (3)	0.008 (3)
C6	0.033 (3)	0.023 (3)	0.036 (3)	0.008 (2)	0.003 (2)	0.005 (2)
C7	0.028 (3)	0.037 (4)	0.041 (4)	0.001 (3)	0.008 (3)	0.003 (3)
C8	0.037 (4)	0.034 (4)	0.031 (3)	0.002 (3)	−0.001 (3)	−0.003 (3)
C9	0.038 (4)	0.028 (3)	0.054 (4)	−0.004 (3)	0.002 (3)	0.001 (3)
C10	0.040 (4)	0.048 (4)	0.056 (4)	−0.008 (3)	−0.003 (3)	0.003 (3)
C11	0.030 (3)	0.056 (4)	0.051 (4)	−0.007 (3)	−0.004 (3)	0.001 (4)
C12	0.028 (3)	0.048 (4)	0.048 (4)	0.006 (3)	−0.008 (3)	−0.001 (3)
C13	0.034 (3)	0.032 (3)	0.029 (3)	−0.001 (2)	0.010 (3)	0.001 (3)

### Geometric parameters (Å, º)

**Table:**

Br1—C2	1.893 (5)	C5—C6	1.442 (7)
Br2—C4	1.902 (5)	C6—C7	1.429 (8)
O1—C1	1.351 (6)	C7—C8	1.476 (8)
O1—C13	1.393 (6)	C8—C13	1.379 (7)
O2—C7	1.262 (7)	C8—C9	1.390 (8)
O3—C3	1.331 (7)	C9—C10	1.368 (8)
O3—H3	0.8200	C9—H9	0.9300
O4—C5	1.343 (6)	C10—C11	1.374 (8)
O4—H4	0.8200	C10—H10	0.9300
C1—C2	1.381 (7)	C11—C12	1.368 (8)
C1—C6	1.411 (7)	C11—H11	0.9300
C2—C3	1.398 (7)	C12—C13	1.389 (7)
C3—C4	1.402 (7)	C12—H12	0.9300
C4—C5	1.361 (7)		
			
C1—O1—C13	119.9 (4)	O2—C7—C6	121.3 (5)
C3—O3—H3	109.5	O2—C7—C8	122.7 (5)
C5—O4—H4	109.5	C6—C7—C8	115.9 (5)
O1—C1—C2	117.1 (4)	C13—C8—C9	118.4 (5)
O1—C1—C6	121.4 (5)	C13—C8—C7	119.5 (5)
C2—C1—C6	121.5 (5)	C9—C8—C7	122.2 (5)
C1—C2—C3	120.6 (5)	C10—C9—C8	120.0 (6)
C1—C2—Br1	119.4 (4)	C10—C9—H9	120.0
C3—C2—Br1	120.0 (4)	C8—C9—H9	120.0
O3—C3—C2	124.2 (5)	C9—C10—C11	120.5 (6)
O3—C3—C4	117.3 (5)	C9—C10—H10	119.8
C2—C3—C4	118.5 (5)	C11—C10—H10	119.8
C5—C4—C3	122.1 (5)	C12—C11—C10	121.2 (5)
C5—C4—Br2	119.1 (4)	C12—C11—H11	119.4
C3—C4—Br2	118.8 (4)	C10—C11—H11	119.4
O4—C5—C4	121.2 (5)	C11—C12—C13	117.9 (5)
O4—C5—C6	118.6 (5)	C11—C12—H12	121.1
C4—C5—C6	120.2 (5)	C13—C12—H12	121.1
C1—C6—C7	121.0 (5)	C8—C13—C12	122.0 (5)
C1—C6—C5	117.1 (5)	C8—C13—O1	122.3 (5)
C7—C6—C5	121.9 (5)	C12—C13—O1	115.7 (5)
			
C13—O1—C1—C2	178.1 (5)	O4—C5—C6—C7	1.1 (9)
C13—O1—C1—C6	−1.2 (8)	C4—C5—C6—C7	−178.8 (5)
O1—C1—C2—C3	178.6 (5)	C1—C6—C7—O2	−178.7 (5)
C6—C1—C2—C3	−2.1 (8)	C5—C6—C7—O2	−0.1 (9)
O1—C1—C2—Br1	0.3 (6)	C1—C6—C7—C8	2.1 (8)
C6—C1—C2—Br1	179.6 (4)	C5—C6—C7—C8	−179.2 (5)
C1—C2—C3—O3	−178.2 (5)	O2—C7—C8—C13	179.3 (6)
Br1—C2—C3—O3	0.1 (8)	C6—C7—C8—C13	−1.6 (8)
C1—C2—C3—C4	1.7 (8)	O2—C7—C8—C9	−1.8 (9)
Br1—C2—C3—C4	−180.0 (4)	C6—C7—C8—C9	177.3 (5)
O3—C3—C4—C5	179.3 (5)	C13—C8—C9—C10	−3.0 (9)
C2—C3—C4—C5	−0.6 (9)	C7—C8—C9—C10	178.0 (6)
O3—C3—C4—Br2	1.0 (7)	C8—C9—C10—C11	1.9 (11)
C2—C3—C4—Br2	−178.9 (4)	C9—C10—C11—C12	−1.3 (12)
C3—C4—C5—O4	179.9 (6)	C10—C11—C12—C13	1.9 (10)
Br2—C4—C5—O4	−1.8 (8)	C9—C8—C13—C12	3.7 (9)
C3—C4—C5—C6	−0.2 (9)	C7—C8—C13—C12	−177.4 (6)
Br2—C4—C5—C6	178.1 (4)	C9—C8—C13—O1	−179.2 (6)
O1—C1—C6—C7	−0.8 (8)	C7—C8—C13—O1	−0.3 (9)
C2—C1—C6—C7	179.9 (5)	C11—C12—C13—C8	−3.1 (10)
O1—C1—C6—C5	−179.5 (5)	C11—C12—C13—O1	179.6 (6)
C2—C1—C6—C5	1.2 (8)	C1—O1—C13—C8	1.7 (9)
O4—C5—C6—C1	179.8 (5)	C1—O1—C13—C12	179.0 (5)
C4—C5—C6—C1	−0.1 (8)		

### Hydrogen-bond geometry (Å, º)

**Table:**

*D*—H···*A*	*D*—H	H···*A*	*D*···*A*	*D*—H···*A*
O3—H3···Br1	0.82	2.61	3.139 (5)	124
O4—H4···O2	0.82	1.81	2.555 (7)	149
O3—H3···O2^i^	0.82	2.02	2.741 (7)	147

Symmetry code: (i) −*x*+3/2, *y*+1/2, *z*−1/2.
